# Defining absolute postoperative desmoid risk in familial adenomatous polyposis according to *APC* genotype and family history: retrospective cohort study

**DOI:** 10.1093/bjsopen/zrag090

**Published:** 2026-09-21

**Authors:** Benjamin Zare, Massih Bahar, Susan Clark, Andrew Latchford

**Affiliations:** St Mark’s Centre for Familial Intestinal Cancer, St Mark's Hospital, London, UK; Department of Surgery and Cancer, Imperial College London, London, UK; St Mark’s Centre for Familial Intestinal Cancer, St Mark's Hospital, London, UK; St Mark’s Centre for Familial Intestinal Cancer, St Mark's Hospital, London, UK; Department of Surgery and Cancer, Imperial College London, London, UK; St Mark’s Centre for Familial Intestinal Cancer, St Mark's Hospital, London, UK; Department of Surgery and Cancer, Imperial College London, London, UK

**Keywords:** lower gastrointestinal surgery, desmoid tumour, colectomy, hereditary colorectal cancer

## Abstract

**Background:**

Desmoid disease is a major cause of morbidity and mortality in familial adenomatous polyposis (FAP), particularly after prophylactic colorectal surgery. Although *APC* genotype and family history are recognized risk factors, previous sizeable studies report relative rather than absolute risks. This study aimed to quantify absolute postoperative desmoid risk in FAP patients undergoing risk-reducing colectomy, stratified by *APC* genotype, and to assess the modifying effect of family history.

**Methods:**

This retrospective observational study used records from a prospectively maintained registry. Patients with an *APC* pathogenic variant (PV) 3′ of codon 1399 were classified as high risk, and those with an *APC* PV 5′ of (or at) codon 1399 were classified as low risk. Patients who had not undergone prophylactic colectomy, with < 5 years of postoperative follow-up, or those with a desmoid diagnosed before or at the time of surgery were excluded. Clinical records were reviewed for details of surgery, desmoid diagnosis, and family history. A positive family history was defined as a first-degree relative with genetically confirmed FAP and a diagnosis of desmoid disease (clinical and/or radiological).

**Results:**

Among 48 high-risk patients, 30 (63%) developed desmoid, with no significant difference between colectomy and proctocolectomy (54% *versus* 70%, respectively; χ^2^ = 1.42; *P* = 0.233). Family history was present in 35 of the 48 patients (73%) and increased desmoid risk relative to no family history (80% *versus* 15%, respectively; *P* < 0.01). Among the 1213 low-risk patients, 154 (12.7%) developed desmoids, with no significant effect of surgical procedure (13.9% *versus* 12.1% for proctocolectomy and total/partial colectomy, respectively; χ² = 0.77; *P* = 0.380). Family history increased desmoid risk relative to no family history (30% *versus* 10%, respectively; *P* < 0.01).

**Conclusion:**

A family history of desmoid disease appears to be an important determinant of postoperative desmoid risk in FAP. Although *APC* PV 3′ of codon 1399 does confer a high overall risk, this is largely confined to individuals with first-degree relatives with desmoid. In the absence of a family history, postoperative desmoid risk appeared relatively low regardless of genotype. This supports a more individualized approach to perioperative counselling and surgical decision-making in patients with FAP. In addition, these data highlight that patients with both a 3′ *APC* PV and a positive family history are a particularly high-risk subgroup who may represent an appropriate target population for future chemoprevention trials.

## Teaser

This retrospective study quantified absolute postoperative desmoid risk in familial adenomatous polyposis patients undergoing prophylactic colectomy, stratified by *APC* genotype and family history. High-risk genotype patients showed a 63% desmoid incidence, *versus* 13% in low-risk patients, but family history was the strongest modifier, increasing risk substantially in both high- and low-risk groups compared with no family history (high-risk group, 80% *versus* 15%, respectively; low-risk group, 30% *versus* 10%, respectively). In the absence of a family history, desmoid risk was similar regardless of genotype, supporting more individualized surgical counselling and identifying a highest-risk subgroup for targeted prevention strategies.

## Introduction

Desmoid disease is a rare pathology, accounting for fewer than 3% of soft tissue lesions overall^1^. It is characterized by a clonal proliferation of well differentiated fibroblasts and myofibroblasts. Although most desmoids occur sporadically, the incidence is markedly increased in patients with familial adenomatous polyposis (FAP), an autosomal dominant disorder caused by a pathogenic variant (PV) in the *APC* gene. FAP is characterized by the development of multiple colorectal adenomas, but is also associated with a range of extracolonic manifestations (desmoid among them). The incidence of desmoid disease in patients with FAP is approximately 850-fold higher than in the general population, with a reported lifetime risk of 10–30%^2,3^.

Desmoid is a major source of mortality in FAP. Since the introduction of risk-reducing large bowel resection, several studies^4–6^ have identified desmoid as either the second- or third-leading disease-related cause of death, the others being colorectal cancer and duodenal cancer. In addition, patients with FAP who develop an associated desmoid report a relatively lower health-related quality of life due to a perceived lack of available interventions and a sense of uncertainty associated with the diagnosis^7^.

Many factors have been implicated in the aetiology and risk of desmoid formation in FAP. Trauma, particularly that due to surgery, is important, with at least 80% of desmoids developing after colectomy^8^.

There is a well recognized genotype–phenotype correlation in FAP, with *APC* PVs occurring in different regions of the gene conferring an increased likelihood of specific manifestations^9^. With respect to desmoid disease, Hodgson *et al*.^10^ first reported an increased frequency of desmoids in patients with *APC* PVs 3′ of codon 1444. Subsequent studies by Caspari *et al*.^11^ and Wallis *et al*.^12^ demonstrated a higher incidence of desmoid in patients with a PV between codons 1445 and 1578, and between codons 1395 and 1493, respectively. In 2001, in multivariate analysis, Bertario *et al*.^8^ showed that mutations 3′ of codon 1444 were independent predictors of desmoid development (odds ratio 6.2; 95% confidence interval (c.i.) 2.5 to 15.8). Sinha *et al*.^13^ went on to report that an *APC* PV 3′ of codon 1399 is the strongest risk factor for development of an intra-abdominal desmoid (hazard ratio 5.2; 95% c.i. 2.1 to 13.3; *P* = 0.001), defining this as high-risk far 3′ constitutional PV.

Despite this genotype–phenotype correlation, desmoid disease can and does occur in patients with a constitutional PV at the 5′ end of the *APC* gene. In recent years, the relative contribution of all the described factors to the risk of desmoid has started to be refined further. For example, both Bertario *et al*.^8^ and Sturt *et al*.^14^ demonstrated that a positive family history of desmoid (defined as the presence of one or more affected first-degree relatives) is a predictor of desmoid formation independent of underlying genotype. Despite this progress, the absolute risk of desmoid development conferred by each of the major risk factors (alone and in combination) has yet to be reported.

The aim of this study was to quantify the absolute risk of desmoid development after large bowel resection conferred by family history within the context of operative management and *APC* genotype to improve the clinical management and counselling of patients with FAP through more informed risk stratification.

## Methods

Patients with a high-risk *APC* variant (defined as a constitutional PV 3′ of codon 1399) and those with a low-risk variant (defined as a constitutional PV at or 5′ of codon 1399) were identified retrospectively in March 2025 from a prospectively maintained polyposis registry at St Mark's Hospital. Patients who had not undergone prophylactic colonic surgery and those with less than 5 years of postoperative follow-up were excluded, as were patients for whom no surgical details were available. All patients who had undergone prophylactic colonic surgery within the St Mark's Hospital Polyposis Registry were included, including patients who underwent surgery at outside institutions. The rationale for excluding patients with less than 5 years of follow-up was based on collated evidence indicating that most FAP-associated desmoids develop within 5 years of surgery, with a median time to onset of 2.1–4.7 years after surgery^15^. Thus, the inclusion of patients with a shorter follow-up could lead to underestimation of desmoid incidence by misclassifying patients as unaffected before they have reached the typical window for desmoid development.

Within each genotype group, medical records were reviewed to obtain data on surgical history, desmoid diagnosis, and family history of desmoid disease. Desmoid diagnosis was confirmed clinically (that is, by physical examination) and/or radiologically, acknowledging that routine systematic postoperative surveillance is not performed by St Mark's Hospital (and therefore clinically insignificant desmoids may be overlooked). Patients whose desmoid was diagnosed before surgery or at the time of surgery were excluded. Patients with desmoid precursor lesions (which are almost exclusively detected during surgery and by those accustomed to their subtle appearance) were not excluded because, although patients with precursor lesions who underwent surgery at St Mark's Hospital (which were relatively few) could be reliably identified, this was not possible for patients who, while under care of St Mark's Hospital, underwent colonic surgery at other centres. For the purposes of the present study, a positive family history of FAP-associated desmoid was defined as the presence of a first degree relative with a genetically confirmed diagnosis of FAP (confirmed *APC* PV) as well as a clinically or radiologically confirmed diagnosis of desmoid. Patients with unknown desmoid family history (very few, reflecting the maturity of this registry established over 100 years ago) were classified as having no family history to avoid inflation of the estimated effect size of this established risk factor in the analysis of absolute desmoid risk.

Under UK National Health Service (NHS) Research Ethics Committee criteria, this project met the definition of a clinical service evaluation rather than research and, as such, did not require formal ethics approval. The evaluation was registered with the clinical audit team at London North West University Hospitals NHS Trust.

Statistical analyses were performed using categorical data comparisons, with proportions compared using Pearson's χ^2^ test or Fisher's exact test where appropriate. All tests were two-sided, with statistical significance defined as *P* < 0.05. All statistical analyses were performed using Stata^®^ version 18.0 (StataCorp, College Station, TX, USA). Missing data were minimal; patients with unavailable surgical details were excluded from the analysis, whereas patients with an unknown family history of desmoid disease were classified as having no family history, as described above, to avoid inflation of the estimated effect size associated with this established risk factor.

A specialist polyposis patient support group was involved in the design and dissemination plans for this research.

## Results

### High-risk genotype

**Fig. 1:**
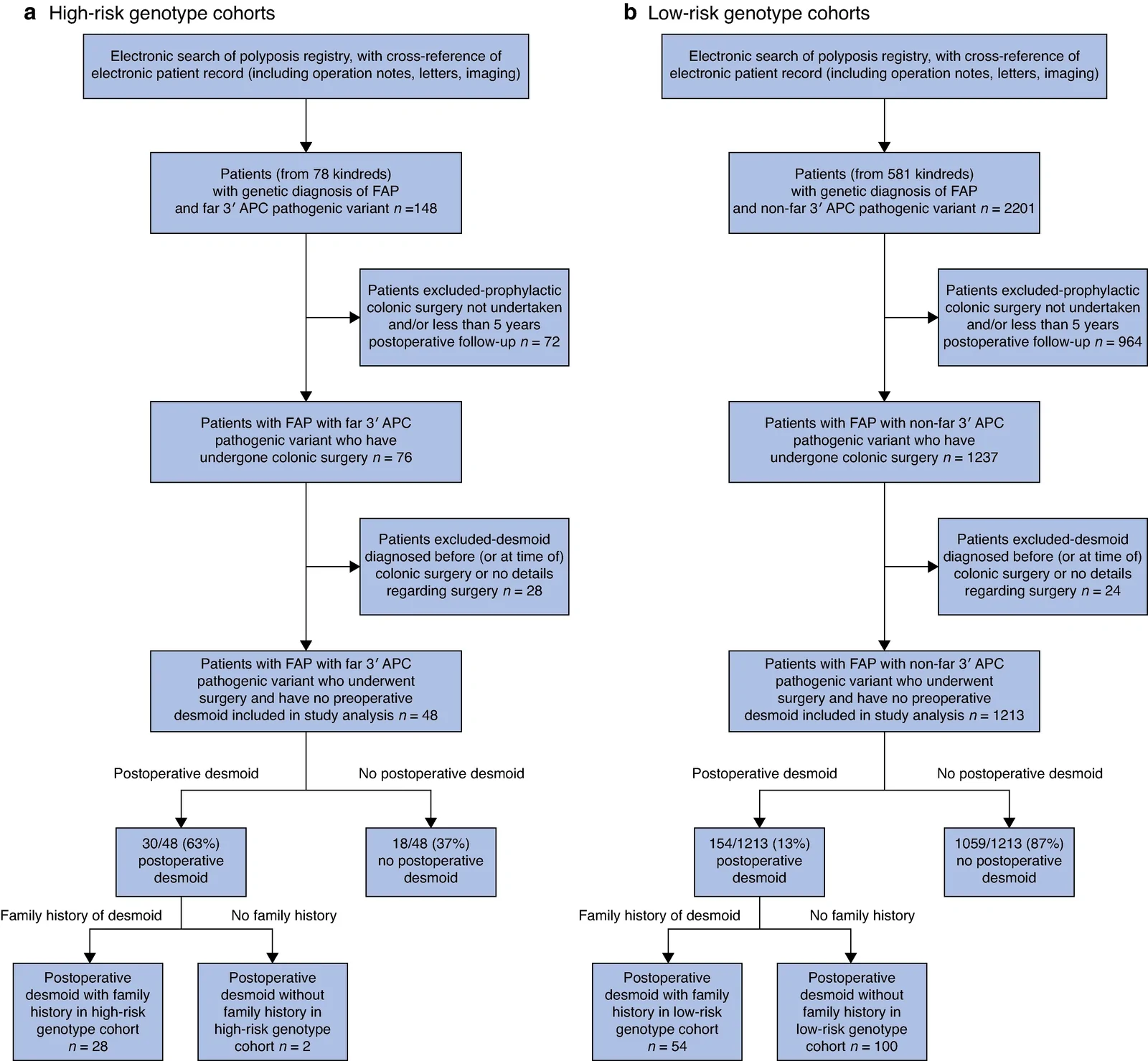
Flow charts showing study identification and exclusion Study identification and exclusion in **a** high-risk and **b** low-risk genotype cohorts. FAP, familial adenomatous polyposis. (a) Study flow chart for patients with high-risk *APC* genotype, defined as a pathogenic variant 3′ of codon 1399. (b) Study flow chart for patients with low-risk *APC* genotype, defined as a pathogenic variant at or 5′ of codon 1399.

The high-risk genotype group initially comprised 148 patients (from 78 kindreds). Of these 148 patients, 72 (48.6%) had an intact large bowel and were excluded. In another 28 patients, the desmoid was diagnosed before or during surgery, or details regarding the surgery were not available, so these patients were also excluded. Thus, the final high-risk cohort consisted of 48 patients (Fig 1a).

Among the 48 high-risk genotype patients, desmoid developed in 30 (63%) after surgery. The surgical intervention was total colectomy with either ileo–rectal anastomosis or ileo–distal sigmoidal anastomosis in 24 patients (50%) and proctocolectomy in the remaining 24 patients (50%). The risk of postoperative desmoid did not differ with surgical intervention, with 13 (54%) of those undergoing total colectomy and 17 (70%) of those undergoing proctocolectomy developing desmoid (χ^2^ = 1.42; *P* = 0.233).

**Fig. 2:**
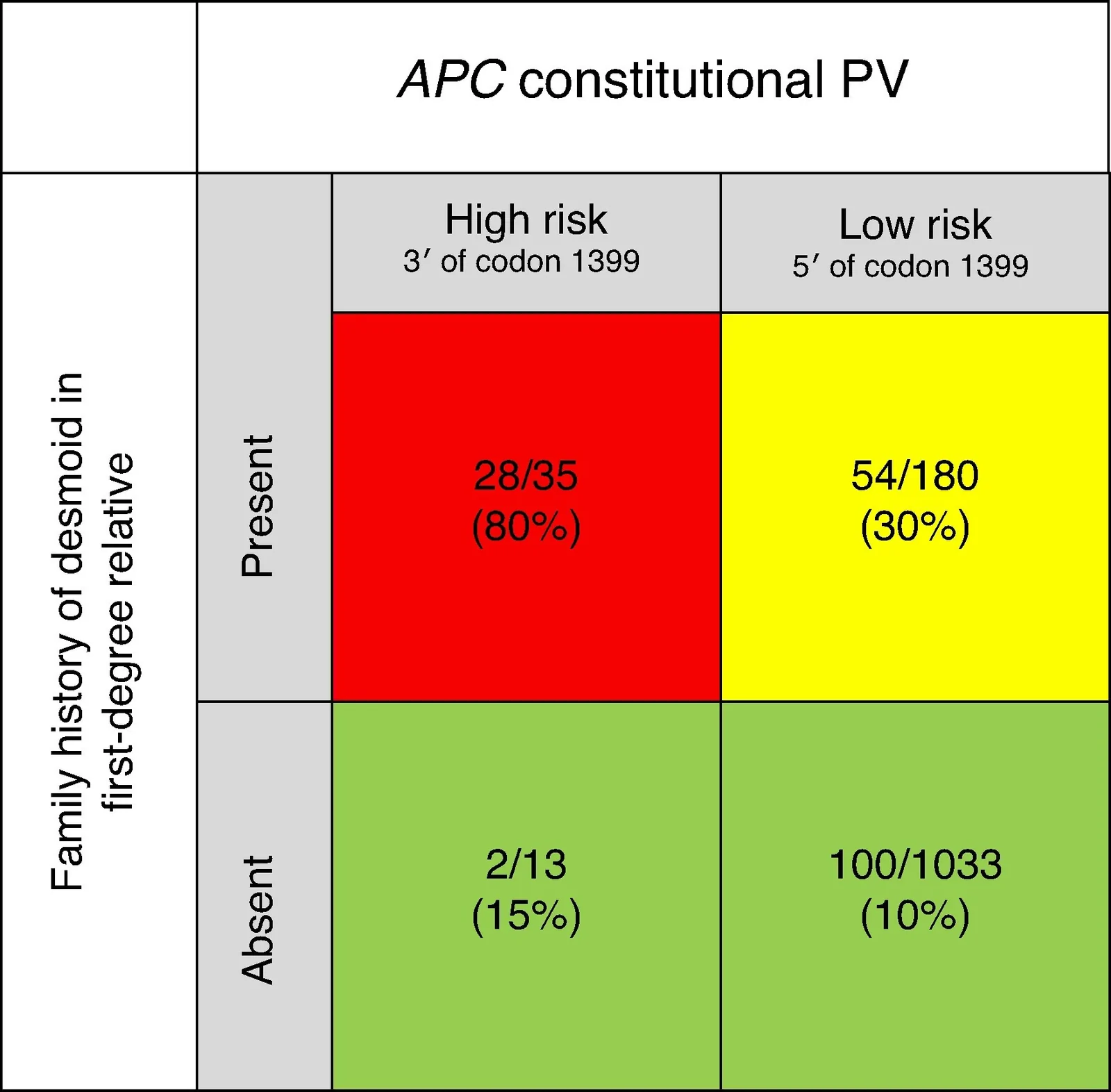
Risk table for predicting the absolute risk of postoperative desmoid occurrence for pathogenic variant patients after prophylactic colonic surgery The absolute risk is based on the *APC* constitutional PV (3′ or 5′ of codon 1399) and a family history of desmoid in a first-degree relative (present or absent). PV, pathogenic variant. Risk was highest in patients with an *APC* pathogenic variant 3′ of codon 1399 and a family history of desmoid disease (28/35; 80%), compared with 15% without family history. In patients with an *APC* variant at or 5′ of codon 1399, risk was 30% with family history and 10% without family history.

A family history of desmoid was present in 35 of the 48 patients (73%) in the high-risk genotype cohort. Of the 35 patients with a family history, 28 (80%) developed postoperative desmoid, compared with 2 patients (15%) among those with no family history (χ^2^ = 14.1; *P* < 0.01; *Figs 1a*, *2*).

### Low-risk genotype

In the low-risk group, 2201 patients (from 581 kindreds) were initially identified. No surgery had been performed in 964 patients. Desmoid was diagnosed before surgical intervention or at the time of surgery in 24 patients, leaving a final cohort of 1213 patients in the low-risk group (Fig 1b).

The primary operations in the low-risk group were proctocolectomy in 388 patients (32.0%), 271 of whom underwent restorative proctocolectomy, colectomy in 811 patients (66.9%), and partial/segmental colonic resection in 14 patients (1.2%). In total, 154 patients (12.7%) in the low-risk group developed desmoid after surgery. The risk of postoperative desmoid did not differ between types of surgery, with 54 patients (13.9%) who underwent proctocolectomy and 100 patients (12.1%) who underwent total/partial colectomy developing desmoid (χ^2^ = 0.77; *P* = 0.380).

A family history of desmoid was present in 180 patients (14.8%) in the low-risk cohort. Of these 180 patients, 54 (30.0%) developed postoperative desmoid, compared with 100 of 1033 patients (9.7%) with no family history (χ^2^ = 57.1; *P* < 0.01; *Figs 1b*, *2*).

## Discussion

This study provides a detailed quantification of the absolute risk of desmoid development in patients with FAP after risk-reducing large bowel resection, stratified by *APC* genotype and family history. Through analysis of a sizeable prospectively maintained registry and application of stringent inclusion criteria, the findings of this study refine existing genotype–phenotype associations and translate relative risks into clinically meaningful absolute risks that can directly inform patient counselling and management. The findings show that family history is an important discriminator of postoperative desmoid risk. In the absence of a family history, postoperative desmoid risk appeared relatively low regardless of genotype, an observation with important implications for clinical management.

Consistent with previous genotype–phenotype studies, patients harbouring an *APC* PV 3′ of codon 1399 exhibited an elevated overall risk of desmoid formation. Earlier studies^8,10–13^ established the association between distal *APC* PVs and desmoid development, largely through relative risk estimates. Similarly, others^16,17^ demonstrated the site of PV as a major predictor of desmoid disease, particularly intra-abdominal desmoid. However, absolute postoperative risks were not defined. In the present cohort, approximately 63% of surgically treated patients with a far 3′ PV developed desmoid disease, confirming that this genotype confers a very high baseline susceptibility following operative trauma^14^.

Importantly, however, genotype alone does not adequately define risk. An important observation in this study was the magnitude of the effect attributable to family history. In patients with a far 3′ PV, the presence of a first-degree relative with desmoid increased the absolute postoperative risk to 80%, compared with only 15% in patients without such a history (χ^2^ = 14.1; *P* < 0.01). Thus, within the high-risk genotype group, family history stratified patients into two clinically distinct populations: one with extremely high penetrance of postoperative desmoid, and another with comparatively modest risk.

A similarly powerful modifying effect of family history was observed in the low-risk genotype group (*APC* PV 5′ of codon 1399). Although the overall postoperative desmoid risk in this group was 13%, the presence of a positive family history increased risk threefold to 30%, compared with 10% in those without a family history (χ^2^ = 57.1; *P* < 0.01). These findings corroborate earlier reports^8,14^, which demonstrated that family history predicts desmoid independently of genotype. The data from the present study extend those observations by quantifying the magnitude of this effect in absolute terms using an especially sizeable cohort and, crucially, by demonstrating its consistency across variant groups.

Perhaps the most clinically significant observation of this study is that in the absence of a family history, postoperative desmoid risk was low and broadly comparable regardless of genotype: 15% in far 3′ PV carriers and 10% in 5′ PV carriers. This suggests that genotype alone may not adequately stratify desmoid risk. Although distal *APC* variants undoubtedly confer increased susceptibility at the population level, the absence of familial clustering appears to mitigate this risk substantially. From a clinical perspective, this suggests that patients with a far 3′ PV but no family history may not warrant the same intensity of concern, surveillance, or pre-emptive counselling as those in whom both risk factors co-exist. Conversely, even patients with so-called low-risk genotypes merit heightened attention if they have a positive family history.

These observations support the hypothesis that additional heritable modifiers beyond *APC* PV contribute to desmoid pathogenesis. The marked intrafamilial aggregation of disease (over and above genotype) suggests the presence of other factors (such as genetic modifiers, epigenetic influences, hormonal factors, or shared environmental exposures) that may modulate desmoid development. The identification of such modifiers remains an important area for future investigation.

With respect to surgery, the findings of the present study align with those of the meta-analysis by Aelvoet *et al*.^18^, who demonstrated no significant difference in desmoid risk between proctocolectomy and colectomy. This supports the concept that surgical trauma, rather than the extent of resection, is the principal operative trigger. Although the exclusion of patients with preoperative or intraoperative desmoid strengthens the attribution of risk to postsurgical events, the present study was not specifically powered to examine more granular operative variables (including surgical approach), which have been explored in more recent analyses^19–21^.

Several limitations of this study warrant consideration. Despite the size of the overall registry, subgroup analyses, particularly within the high-risk genotype cohort, are limited by relatively small numbers. In addition, the retrospective nature of data extraction may have introduced ascertainment bias, although the use of a prospectively maintained registry and strict objective diagnostic criteria mitigate this risk. The inclusion of patients treated initially at outside institutions may have introduced referral bias, because patients developing desmoid disease may have been preferentially referred to St Mark's Hospital as a tertiary FAP centre. Otherwise, routine systematic postoperative surveillance for desmoid is not performed by St Mark's Hospital (in keeping with practice in most other specialist centres). With imaging and/or physical examination for possible desmoid triggered by potentially clinically consistent symptoms, some asymptomatic or radiologically occult desmoids may have remained undetected. It was not possible to account for current or previous medication (such as non-steroidal anti-inflammatory drugs and oestrogen for example through the oral contraceptive pill) administration, nor was it possible to address hormonal factors; hypothetically, any of these factors may affect desmoid incidence, although the evidence base for their respective effects on desmoid disease development is limited.

Together, the findings of the present study redefine the hierarchy of risk factors for desmoid disease in FAP. Although genotype remains important, family history appears to be a major modifier of absolute postoperative risk, and its absence identifies a group of patients with relatively low risk regardless of the site of the PV. This has direct implications for discussions regarding surgical timing, informed consent, postoperative counselling, and the design of preventive strategies.

## Supplementary Material

zrag090_Supplementary_Data

## Data Availability

The data sets generated/analysed during the present study are not publicly available due to institutional and patient privacy restrictions but are available in deidentified form from the corresponding author upon reasonable request and with permission of London North West University Hospitals NHS Trust. Proposals for access should be sent to b.zare@nhs.net.
