# Supplementary material for: Defining absolute postoperative desmoid risk in familial adenomatous polyposis according to APC genotype and family history: retrospective cohort study
Source: BJS Open. 2026 Sep 21;10(5):zrag090. doi: 10.1093/bjsopen/zrag090 (PMC13591399; doi:10.1093/bjsopen/zrag090)
Supplement: Supplementary file 1 [file zrag090_supplementary_data.docx]

**Defining absolute postoperative desmoid risk in familial adenomatous polyposis according to APC genotype and family history: Retrospective cohort study**

Authors

Benjamin Zare^1,2^, Massih Bahar^1^, Susan Clark^1,2^, Andrew Latchford^1,2^

^1^ St Mark’s Centre for Familial Intestinal Cancer, St Mark's Hospital, London, UK.

^2^ Department of Surgery and Cancer, Imperial College London, London, UK.

**Corresponding author.** Benjamin Zare **Corresponding author address:** St Mark’s Hospital, Acton Lane, London NW10 7NS

**ORCID ID** 0000-0002-8866-3209; **Twitter** @zare_benjamin

**Supplementary Materials - Index**

| **Supplementary Methods** |  |
| --- | --- |
| Detail | *N/A* |
| Detail | *N/A* |
| **Supplementary Results** |  |
| Detail | *N/A* |
| Detail | *N/A* |
| **Supplementary Appendixes** |  |
| Detail | *N/A* |
| Detail | *N/A* |
| **Supplementary Figures and Tables** |  |
| Detail | *N/A* |
| Detail | *N/A* |
| **References** | *N/A* |
| **STROBE checklist** | *page 2* |

**STROBE Statement-checklist of items that should be included in reports of observational studies**

*Manuscript: Defining absolute postoperative desmoid risk in familial adenomatous polyposis according to APC genotype and family history: Retrospective cohort study*

|  | **Item No.** | **Recommendation** | **Page No.** | **Relevant text from manuscript** |
| --- | --- | --- | --- | --- |
| Title and abstract | 1 | (a) Indicate the study’s design with a commonly used term in the title or the abstract | 1 | The title states: “Retrospective cohort study”. The abstract Methods also states: “Retrospective observational study using records from a prospectively maintained registry.” |
|  |  | (b) Provide in the abstract an informative and balanced summary of what was done and what was found | 1-2 | The abstract summarises the background, aim, methods, key results, and conclusions. It reports the genotype groups, exclusions, family-history definition, absolute postoperative desmoid risks, and the conclusion that family history is an important determinant of postoperative desmoid risk. |
| **Introduction** |  |  |  |  |
| Background/rationale | 2 | Explain the scientific background and rationale for the investigation being reported | 2-4 | The Introduction explains that desmoid disease is an important cause of morbidity and mortality in FAP, is associated with surgery and *APC* genotype, and that previous sizeable studies have generally reported relative rather than absolute risks. It identifies the need to quantify absolute risk according to genotype and family history. |
| Objectives | 3 | State specific objectives, including any prespecified hypotheses | 4 | The stated aim was “to quantify the absolute risk of desmoid development following large bowel resection conferred by family history within the context of operative management and *APC* genotype,” to improve counselling and risk stratification. |
| **Methods** |  |  |  |  |
| Study design | 4 | Present key elements of study design early in the paper | 4 | The Methods state that patients were identified retrospectively from a prospectively maintained polyposis registry. The abstract also describes the work as a retrospective observational study. |
| Setting | 5 | Describe the setting, locations, and relevant dates, including periods of recruitment, exposure, follow-up, and data collection | 4-5 | Patients were identified from the St Mark’s prospectively maintained polyposis registry in March 2025. Included patients had undergone prophylactic colonic surgery, including surgery performed at outside institutions, and required at least 5 years of postoperative follow-up. |
| Participants | 6 | (a) Cohort study - Give the eligibility criteria, and the sources and methods of selection of participants. Describe methods of follow-up Case-control study - Give the eligibility criteria, and the sources and methods of case ascertainment and control selection. Give the rationale for the choice of cases and controls Cross-sectional study - Give the eligibility criteria, and the sources and methods of selection of participants | 4-5 | Eligible patients had FAP with an *APC* pathogenic variant, were classified by *APC* PV position relative to codon 1399, and had undergone prophylactic colonic surgery. Patients without prophylactic colonic surgery, with less than 5 years’ postoperative follow-up, with desmoid diagnosed before or at surgery, or with unavailable surgical details were excluded. Clinical records were reviewed for surgery, desmoid diagnosis, and family history. |
|  |  | (b) Cohort study - For matched studies, give matching criteria and number of exposed and unexposed Case-control study - For matched studies, give matching criteria and the number of controls per case |  | N/A - not a matched cohort study. |
| Variables | 7 | Clearly define all outcomes, exposures, predictors, potential confounders, and effect modifiers. Give diagnostic criteria, if applicable | 4-5 | The main outcome was postoperative desmoid disease, diagnosed clinically and/or radiologically. Main exposures/predictors were *APC* genotype group (PV 3′ of codon 1399 versus at/5′ of codon 1399), family history of FAP-associated desmoid in a first-degree relative, and type of prophylactic colonic surgery. |
| Data sources/measurement | 8* | For each variable of interest, give sources of data and details of methods of assessment (measurement). Describe comparability of assessment methods if there is more than one group | 4-5 | Data came from the prospectively maintained polyposis registry and review of clinical records. Desmoid diagnosis was confirmed clinically and/or radiologically; the manuscript notes that routine systematic postoperative surveillance is not carried out, so clinically insignificant desmoids may be overlooked. |
| Bias | 9 | Describe any efforts to address potential sources of bias | 4-5, 9 | Eligibility required at least 5 years of postoperative follow-up to avoid underestimating desmoid incidence. Patients with desmoid before or at surgery were excluded. Patients with unavailable surgical details were excluded. Unknown family history was classified as no family history to avoid inflating the estimated effect of family history. The Discussion acknowledges ascertainment bias, referral bias, lack of systematic surveillance, and unmeasured medication/hormonal factors. |
| Study size | 10 | Explain how the study size was arrived at | 6-7 | All eligible patients in the registry were included. The high-risk group began with 148 patients; after exclusions, 48 were analysed. The low-risk group began with 2201 patients; after excluding 964 without surgery and 24 with pre-/intra-operative desmoid, 1213 were analysed. |
| Quantitative variables | 11 | Explain how quantitative variables were handled in the analyses. If applicable, describe which groupings were chosen and why | 4-5 | Variables were analysed categorically. *APC* PV location was grouped as high risk (3′ of codon 1399) or low risk (at or 5′ of codon 1399). Family history was grouped as present or absent. Surgery was grouped as colectomy/proctocolectomy in the high-risk cohort and proctocolectomy/colectomy/partial or segmental colonic resection in the low-risk cohort. |
| Statistical methods | 12 | (a) Describe all statistical methods, including those used to control for confounding | 5 | Categorical data comparisons were performed using Pearson’s chi-squared test or Fisher’s exact test where appropriate. Tests were two-sided, statistical significance was *p* < 0.05, and analyses were performed in Stata version 18.0. No multivariable adjustment is reported. |
|  |  | (b) Describe any methods used to examine subgroups and interactions | 6-7 | Analyses were stratified by *APC* genotype group, family history of desmoid disease, and type of surgical intervention. No formal interaction testing is explicitly reported. |
|  |  | (c) Explain how missing data were addressed | 5 | Patients with unavailable surgical details were excluded. Patients with unknown family history of desmoid disease were classified as having no family history, as prespecified, to avoid inflation of the estimated effect associated with family history. |
|  |  | (d) Cohort study - If applicable, explain how loss to follow-up was addressed Case-control study - If applicable, explain how matching of cases and controls was addressed Cross-sectional study - If applicable, describe analytical methods taking account of sampling strategy | 4-5 | Eligibility required at least 5 years of postoperative follow-up. Patients with less than 5 years’ postoperative follow-up were excluded. |
|  |  | (e) Describe any sensitivity analyses |  | N/A - no sensitivity analyses are explicitly reported. |
| **Results** |  |  |  |  |
| Participants | 13* | (a) Report numbers of individuals at each stage of study - eg numbers potentially eligible, examined for eligibility, confirmed eligible, included in the study, completing follow-up, and analysed | 6-7, 10 | High-risk genotype: 148 patients from 78 kindreds were identified; 72 with intact large bowel and 28 with pre-/intra-operative desmoid or unavailable surgery details were excluded; 48 were analysed. Low-risk genotype: 2201 patients from 581 kindreds were identified; 964 without surgery and 24 with pre-/intra-operative desmoid were excluded; 1213 were analysed. Figure 1 provides the flow chart. |
|  |  | (b) Give reasons for non-participation at each stage | 6-7 | Reasons for exclusion included no prophylactic colonic surgery/intact large bowel, desmoid diagnosed before or at surgery, unavailable surgical details, and less than 5 years of postoperative follow-up as specified in the Methods. |
|  |  | (c) Consider use of a flow diagram | 10 | Figure 1 is described as a flow chart of study identification and exclusion in the high-risk and low-risk genotype cohorts. |
| Descriptive data | 14* | (a) Give characteristics of study participants (eg demographic, clinical, social) and information on exposures and potential confounders | 6-7 | The Results describe genotype cohort size and kindreds, surgical procedure distribution, family-history status, and desmoid outcomes. High-risk cohort: 48 patients, with 24 colectomy/IDSA-IRA and 24 proctocolectomy; 35/48 had family history. Low-risk cohort: 1213 patients, with 388 proctocolectomy, 811 colectomy, and 24 partial/segmental resection; 180/1213 had family history. |
|  |  | (b) Indicate number of participants with missing data for each variable of interest | 5-7 | Patients with unavailable surgical details were excluded. The high-risk Results combine patients with pre-/intra-operative desmoid or unavailable surgery details in one excluded group of 28. The manuscript states that missing data were minimal and that unknown family history was classified as no family history. |
|  |  | (c) Cohort study - Summarise follow-up time (eg, average and total amount) | 4-5 | A minimum of 5 years’ postoperative follow-up was required. The manuscript does not report median, mean, or total follow-up time. |
| Outcome data | 15* | Cohort study - Report numbers of outcome events or summary measures over time | 6-7 | Postoperative desmoid developed in 30/48 (63%) high-risk genotype patients and 154/1213 (13%) low-risk genotype patients. Within high-risk patients, risk was 28/35 (80%) with family history versus 2/13 (15%) without. Within low-risk patients, risk was 54/180 (30%) with family history versus 100/1033 (10%) without. |
|  |  | Case-control study - Report numbers in each exposure category, or summary measures of exposure |  | N/A - not a case-control study. |
|  |  | Cross-sectional study - Report numbers of outcome events or summary measures |  | N/A - not a cross-sectional study. |
| Main results | 16 | (a) Give unadjusted estimates and, if applicable, confounder-adjusted estimates and their precision (eg, 95% confidence interval). Make clear which confounders were adjusted for and why they were included | 6-7 | Unadjusted absolute risks are reported. High-risk genotype: 30/48 (63%) developed desmoid; family history increased risk from 15% to 80% (chi-squared = 14.1; p < 0.01). Low-risk genotype: 154/1213 (13%) developed desmoid; family history increased risk from 10% to 30% (chi-squared = 57.1; p < 0.01). No confounder-adjusted estimates are reported. |
|  |  | (b) Report category boundaries when continuous variables were categorized | 4-5 | *APC* PV group boundaries were defined by codon 1399: high-risk = PV 3′ of codon 1399; low-risk = PV at or 5′ of codon 1399. Positive family history was defined as a first-degree relative with genetically confirmed FAP and clinically or radiologically confirmed desmoid disease. |
|  |  | (c) If relevant, consider translating estimates of relative risk into absolute risk for a meaningful time period | 6-7, 11 | The manuscript focuses on absolute postoperative risk. Figure 2 presents a risk table for postoperative desmoid occurrence after prophylactic colonic surgery according to *APC* PV location and family history. |
| Other analyses | 17 | Report other analyses done - eg analyses of subgroups and interactions, and sensitivity analyses | 6-7 | Subgroup analyses compared desmoid risk by surgical procedure and family history within high-risk and low-risk genotype cohorts. No significant effect of surgical procedure was found in either cohort; family history was significantly associated with postoperative desmoid risk in both cohorts. No sensitivity analyses are explicitly reported. |
| **Discussion** |  |  |  |  |
| Key results | 18 | Summarise key results with reference to study objectives | 7-9 | The Discussion states that the study quantified absolute postoperative desmoid risk by *APC* genotype and family history, and found family history to be the strongest discriminator of risk. It highlights risks of 80% versus 15% in high-risk genotype patients with versus without family history, and 30% versus 10% in low-risk genotype patients with versus without family history. |
| Limitations | 19 | Discuss limitations of the study, taking into account sources of potential bias or imprecision. Discuss both direction and magnitude of any potential bias | 9 | The Discussion acknowledges small subgroup numbers in the high-risk cohort, retrospective extraction, possible ascertainment bias, referral bias, absence of routine systematic postoperative surveillance, possible missed asymptomatic/radiologically occult desmoids, and inability to account for medications, oestrogen exposure, hormonal factors, or other potential influences. |
| Interpretation | 20 | Give a cautious overall interpretation of results considering objectives, limitations, multiplicity of analyses, results from similar studies, and other relevant evidence | 7-10 | The authors interpret that genotype remains important but family history appears to be a major modifier of absolute postoperative desmoid risk. They relate findings to prior genotype-phenotype and family-history studies and conclude that patients with both far 3′ APC PV and positive family history represent a particularly high-risk group, while absence of family history identifies a relatively lower-risk group. |
| Generalisability | 21 | Discuss the generalisability (external validity) of the study results | 9 | Generalisability is addressed indirectly. The manuscript notes inclusion of a sizeable specialist registry and patients who underwent surgery both at St Mark’s and outside institutions, but also acknowledges possible referral bias from a tertiary FAP centre and lack of routine surveillance, which may limit external validity. |
| **Other information** |  |  |  |  |
| Funding | 22 | Give the source of funding and the role of the funders for the present study and, if applicable, for the original study on which the present article is based | 12 | The Funding statement says: “The authors have not declared a specific grant for this research from any funding agency in the public, commercial or not-for-profit sectors.” |
|  |  | *Give information separately for cases and controls in case-control studies and, if applicable, for exposed and unexposed groups in cohort and cross-sectional studies. |  |  |
